# Genomic and Functional Characterization of *Enterococcus mundtii* QAUEM2808, Isolated From Artisanal Fermented Milk Product Dahi

**DOI:** 10.3389/fmicb.2019.00434

**Published:** 2019-03-26

**Authors:** Farah Nawaz, Muhammad Nadeem Khan, Aqib Javed, Ibrar Ahmed, Naeem Ali, Muhammad Ishtiaq Ali, Syeda Mariam Bakhtiar, Muhammad Imran

**Affiliations:** ^1^Department of Microbiology, Faculty of Biological Sciences, Quaid-i-Azam University, Islamabad, Pakistan; ^2^Alpha Genomics Private Limited, Islamabad, Pakistan; ^3^Department of Bioinformatics and Biosciences, Capital University of Science and Technology, Islamabad, Pakistan

**Keywords:** *Enterococcus mundtii*, dairy fermentation, antibiotic resistance, antagonism, bacteriocins, probiotics, food safety, adjunct culture

## Abstract

Microbial strains with a unique combination of technological and bioactive properties are preferred for industrial applications. The present study was conducted to evaluate the potential use of *Enterococcus mundtii* QAUEM2808 (NCBI Accession Number: LSMC00000000) in milk fermentation. This strain was isolated from Dahi, an indigenous fermented milk product of South-East Asia. The *in vitro* study confirmed the acidification ability as well as the proteolytic, cellulolytic, and amylolytic enzyme activities of this strain. It also produced a substantial amount of the folate in laboratory media and no physiological dysfunctions in laboratory animals was observed in feeding trials. All these properties were confirmed by *in silico* genome analysis. The *Enterococcus mundtii* QAUEM2808 genome consisted of a single, circular chromosome comprising 2,957,300-bp, 2,587 genes with GC content of 38.5%. Moreover, 16t RNAs, 1, 3 (16S, 23S) rRNAs, 4 ncRNAs, and 91 pseudo genes were also predicted. The majority of genome encode genes for protein, amino acids, carbohydrate, cell wall DNA and RNA metabolisms including all genes required for conversion of lactose to lactic acid. It also exhibited antimicrobial activity against *E. coli* ATCC 10536, *S. aureus* ATCC 6538, *P. aeruginosa* ATCC 9027, and *L. monocytogenes* ATCC 13932 and was found to be sensitive to commonly used antibiotics. The *in silico* analysis revealed the presence of genes for mundaticin and enterocin production, and CRISPER regions, however, the genes for antibiotic resistance were absent. No genes related to the pathogenicity island and prophages were detected by genome mining. Therefore, it could be inferened that *Enterococcus mundtii* QAUEM2808 has the potential to be used in milk fermentation as adjunct culture.

## Introduction

Enterococci have evolved over ages as vastly amended members of the intestinal microbiota of a wide range of hosts and environments like water, soil, fermented foods, and dairy products. In dairy and food ecosystems, Enterococci perform dynamic functions resulting in fermentation and preservation of the food (Dinleyici et al., 2013). Due to their enhanced proteolytic, lipolytic, amylolytic, citrate utilization (Foulquié Moreno et al., 2006), and acidification abilities (Giraffa, 2003), they contribute towards the cheese ripening, aroma and flavor generation. Therefore, many strains have been utilized as primary starter or non-starter secondary cultures in some varieties of cheese (Coppola et al., 1990; Hugas et al., 2003). In dairy products, these are important sources of folate production. Mammalian cells are incapable of synthesizing folate, however, folate is vital for numerous essential cell functions, including metabolism, production of vitamins, amino acids, nucleotides, and cell division (Fox et al., 2015). Deficiency of folate leads to various disorders including birth defects in new born, heart diseases, risk of cancer or Alzheimer's disease (Da Silva et al., 2016). The *Enterococci* are *not generally regarded as safe*, (GRAS), but are well-known for their unique technological, probiotic, and anti-pathogenic attributes (Jamet et al., 2012). Their presence in fermented foods not only improves the sensory profile but also extends the shelf life through production of various bacteriocins (Martín-Platero et al., 2009). Enterocins are an important class of bacteriocins produced by the genus Enterococcus, and are known to have anti pathogenic properties against emerging pathogens in fermented and non-fermented food products (Callewaert et al., 2000; Franz et al., 2007).

Among Enterococci spp, *Enterococcus mundtii* is a ubiquitous bacteria, assigned as a member of group *Enterococcus faecium* on the basis of homology in 16S rDNA sequence (Klein, 2003). It has been associated with raw milk, plants, intestinal tract of humans and dairy cattle (Collins et al., 1986; Giraffa et al., 1997; Giraffa, 2003; Espeche et al., 2009). This bacterium has low GC content ranging between 38 and 39% and lacks catalase and cytochrome-C oxidase enzymes, but can contribute in carbohydrates fermentation to produce lactic acid. It produces enterocins such as Bacteriocin ST15, that are quite active against bacteria such as *Pseudomonas, Clostridium, Klebsiella, Lactobacillus*, and *Acinetobacter* etc. (De Kwaadsteniet et al., 2005; Ferreira et al., 2007; Settanni et al., 2008). It is reported to be used for the prevention of mastitis in cows (Espeche et al., 2009).

Due to increasing consumer's demand for safe and consistent quality of products, there is a growing interest toward the exploration of organisms from the diverse population of wild lactic acid bacteria from dairy sources (Wouters et al., 2002). The emerging genome sequencing technologies made it possible to control the genetics and metabolism of microorganisms (Law, 2001), resulting in greater demand of such functional microorganisms in industrial sectors (De Vuyst, 2000).

In the past few decades *Enterococci* have been considered as opportunistic pathogens, due to the presence of antibiotic resistance and virulence genes within their chromosome or extrachromosomal elements. They are not deemed to be safe, and are considered a sign of fecal contamination and a probable health risk. Hence, studies were restricted only to the clinical isolates of the *Enterococci* spp. However, in the last decade some studies have suggested the role of specific *Enterococcus* strains as probiotics for human and animal use. Some of the *enterococci* strains are supposed to be natural or added starters for fermented products. They are screened for the production of bacteriocin and the ability to act as a starter culture for fermentation (Venema, 2015). Clinical isolates of *Enterococci* spp. showed resistance against gentamycin, imipenem, penicillin, tetracycline, chloramphenicol, erythromycin, lincomycin, and rifampicin (Chingwaru et al., 2003; Çitak et al., 2004). Some *E. faecalis* strains are resistant to antibiotics, particularly to vancomycin and such strains are termed as VRE (Vancomycin resistant Enterococci). Antibiotic resistance is of particular interest in food ecosystems as genes for resistance are mainly present on plasmids, which can be transferred horizontally to other bacterial species (Zanella et al., 2006). Since they also contain antibiotic resistance and virulence genes, scientists are concerned about the safety of *Enterococcus* in food (Giraffa, 2003; Ogier and Serror, 2008).

The virulence of *Enterococcus* spp. is known to be a specific specie trait (Franz et al., 2001; Rice et al., 2003) and resistance against different antibiotics is not enough to classify a strain as virulent. Other traits which contribute to virulence include cell adherence, attacking factors, ability to release toxic substances, and other harmful factors (Eaton and Gasson, 2001; Dogru et al., 2010). Virulence factors are mostly encoded by, or linked with, transposable genomic elements such as plasmids, IS elements, transposons or phages. Large numbers of such elements are present within the Pathogenicity Associated Islands (PAI) (Schmidt and Hensel, 2004). PAIs are specific coding regions in the genome, which are associated with virulence factors (Hacker and Kaper, 2000). Pathogenicity determinants in clinical samples of *Enterococci* are quite significant in comparison to those obtained from animal and food matrix (Ben Omar et al., 2004; Khan et al., 2008). Many strains in *Enterococcus faecium*, including *Enterococcus mundtii*, are reported to produce biogenic amines in food because of their amino acid decarboxylase activity (Kalhotka et al., 2012). The generation of biogenic amines in food is usually considered an undesirable trait; it is used as a quality indicator for food and dairy products (Aldegunde and Mancebo, 2006). Safety evaluation of microbial strains is necessary for them in order to be incorporated in food and it is linked to their use, which includes the dose response mechanism. If the food microorganism remains viable in the intestinal tract of the host, they could lead to potential infection risks (Sanders et al., 2010). Therefore, the recommendation is to properly test the impact of new strains on laboratory animals' physiology and hematology (Shokryazdan et al., 2016).

Here we present the physiological, technological and safety properties of a strain *E. mundtii* QAUEM2808 isolated from fermented milk product, Dahi. Its genome was recently reported in genome announcement (Farah et al., 2016). Our results suggest that this strain is safe and has potential to improve the quality of the product as adjunct culture in milk fermentation.

## Materials and Methods

### Isolation and Physiochemical Analysis

The *E. mundtii* QAUEM2808 was isolated from indigenously fermented milk product Dahi. 10 gram of Dahi sample was homogenized by laboratory vortexing (IKA TTS2, Germany), 100 uL was spreaded on tryptone soya agar (TSA; Oxoid, UK) and then incubated at 37°C for 24 h. Morphological and biochemical characterization of the isolated strain was done by Gram Staining; catalase and oxidase tests.

### Growth Conditions and Technological Characteristics

Growth and acidification capacity of this strain was tested at 15, 30, 37, and 45°C for 72 h in Trypticase Soy Broth (TSB) medium. Tolerance to NaCl was measured by adding 2 and 4% NaCl in TSB basal media while incubating at the same temperatures. Growth index was measured as change in turbidity at 600 nm (Dhillon et al., 2015). Enzymatic potential of the strain was determined in terms of proteolytic, amylolytic, cellulolytic, and lipolytic activities. To determine the proteolytic activity, Milk agar plates (10% w/v; autoclaved at 110°C for 20 min), were streaked with a 24 h old inaculum and were incubated at 37°C for 48 h. Amylolytic activity was determined by streaking nutrient agar plates containing 1% starch with 24 h old culture of *E. mundtii* QAUEM2808 and incubated at 37°C for 48 h. The plates were then swamped with gram's iodine and were left for 15–30 min (Bernfeld, 1955). Carboxymethyl cellulose (CMC) agar (Hankin and Anagnostakis, 1977) media was used to detect cellulose activity of *E. mundtii* QAUEM2808. Autoclaved Tween 80 was added as a lipid substrate in media used for the lipolytic enzymes (Sierra, 1957).

### Screening for Potential Biogenic Amine Production Ability

Potential biogenic amine production was tested by determination of histidine and tyrosine decarboxylation activity, which leads to histamine and tyramine production, respectively. Test was done at 20, 37, and 50°C and the impact of 1, 2, 3, 4, and 5% NaCl concentrations was also measured. Decarboxylation media (pH 5.2) was prepared as described before (Brooks and sodeman, 1974). Testing strain was inoculated in the test tube containing 10 ml of amino acid supplemented TSB and incubated for 48 h. Two control tubes were also incubated under the same conditions: one containing only media supplemented with amino acid (histidine or tyrosine) and the second one containing testing strain in media without amino acid supplementation. Change in media color to purple was noted as positive decarboxylase activity of supplemented amino acid.

### Evaluation of Antibiotic Resistance

Antibiotic resistance was measured by using Kirby and Bauer disc diffusion test (Hudzicki, 2013). After 24 h pre-culturing and culturing in TSB medium, *Enterococcus mundtii* QAUEM2808 was put in test tubes in 2 mL of normal saline (0.9% NaCl) to achieve 0.5M Mac Ferland as turbidity standard. Antibiotics disks Vancomycin (VA) 30 ug, Erythromycin (E) 15 ug, Ciprofloxacin (CIP) 5 ug, Norfloxacin (NOR) 10 ug, piperacillin (PRL) 100 ug, Tazobactum (TZP) 110 ug, Doxycycline (DO) 30 ug, Gentamycin (CN) 10 ug, bacitracin (B) 10 ug and Penicillin (P) 1 IU (Oxoid and Liofilchem) were used in the test. The inhibition zone around the disk was observed after incubating plates for 24 h at 37°C.

### Bacteriocins Production Ability

Bacteriocins production ability was determined by using agar diffusion assay (Yamato et al., 2003). The Cell-free supernatant was obtained by centrifugation of the culture at 8,000 g at 4°C for 10 min, adjusted to final pH of 5.5 with 1 M of NaOH, filtered with 0.45 μm pore size filter and kept at −20°C till use. A volume of 100 uL *Enterococcus mundtii* QAUEM2808, warmed at 37°C for at least 1 h, was suspend in 2.5 mL of soft agar (0.75% MRS) and was poured on an MRS plate to make lawn of indictor microorganism. After solidification, the plate was incubated at 37°C for at least 2.5–3 h. Then 10 μL of supernatant of overnight growing strain of lactic acid bacteria was poured on the filter paper disk. The supernatant containing disk was carefully placed on the lawn of indictor strain. Antimicrobial activity of *Enterococcus mundtii* QAUEM2808 was tested against *L. monocytogenes* (ATCC 13932), *E. coli* (ATCC 10536), S. *aureus* (ATCC 6538), P. *aeruginosa* (ATCC 9027), and *S. epidermidis* (ATCC 12228). The plate was again incubated at 37°C for 24 h. Bacteriocin production was evaluated based on the formation of a clear zone around the disk. The results were expressed in quantitative terms as the diameter (Joensen et al., 2014) of the clear zone around the disk.

### Screening for Folate Production

The standard for folate was obtained from a British pharmacopoeia laboratory, Rawalpindi Pakistan. Different concentrations of powdered standard were made from 250 to 1 ppm solution by dissolving it in phosphate buffer (0.1M, pH8), NaOH and then distilled water was added to make up the volume up to 100 ml. Optical density of all concentrations was analyzed at 600 nm wavelength on spectrophotometer and a standard curve was constructed (Hugenschmidt-Baltzer, 2010). Then *E. mundtii* QAUEM2808 was inoculated in TSB (pH 7.2 ± 0.2, Sigma Aldrich), MRS broth and M17 broth (Merck, Germany) then incubated for 24–48 h at 10, 30, 31.8, 37, and 50°C and folate content was analyzed. For intracellular folate production by strain, 2 ml of culture was taken and sonicated at 50,000 Hz for 2 min by giving pulse of 2 s. The sample was then exposed to heat treatment at 100°C for about 5 min. The purpose of heat treatment was to release any folate bounded with proteins. Cells-free extract was then gained after centrifugation at 10,000 rpm for 10 min. **S**upernatant was taken for OD at 600 nm on the spectrophotometer. The concentration of folate was calculated in ppm from the standard curve of folic acid (Kodi et al., 2015).

### *In—vivo* Safety Assessment

For *in vivo* safety analysis, *Enterococcus mundtii* QAUEM2808 was grown in Tryptic soy broth (TSB; Oxide UK), about 2 × 10^09^ cfu per mice per day was administered for the course of 3 months. Balb/c mice *n* = 15, at the age of 4 weeks were selected for the trial and were dissected at the age of 16 weeks. The mice were housed in separate cages, under standard conditions: equal light-dark cycle and kept at a temperature range of 18–23°C. They were fed non sterile diet (standard animal feed) and water was accessible throughout. The *Enterococcus mundtii* QAUEM2808 was successfully isolated from mice stomach and small intestine after dissection. The sample of Blood was collected in EDTA tubes before mice dissection, after anesthetizion with chloroform. Blood samples were analyzed with XP-300-Hematology-Analyzer (Sysmex, USA). Leukocyte, erythrocyte and platelet count, hemoglobin concentration (HB), mean corpuscular volume (MCV), mean corpuscular hemoglobin (MCH), mean corpuscular hemoglobin concentration (MCHC), red cell distribution width, mean platelet volume, and hematocrit were determined in Blood CP.

### Genome Sequencing and Analysis

The *Enterococcus mundtii* QAUEM2808 DNA was extracted from fresh culture following (He, 2011). Genome was sequenced using a whole-genome shotgun (WGS) approach, using HiSeq 2000 System. Sequence quality was assessed by FastQC (Andrews, 2010). To optimize poor quality, the sequence “Masking” function of Galaxy Server was used. Genome assembly parameters were optimized by Velvet Optimizer. Velvet (Zerbino and Birney, 2008) was used to assemble the draft genome. Draft genome sequence was extracted by Artemis (Rutherford et al., 2000) in FASTA format, which was provided to RAST server for annotation, and the putative gene products were identified (Aziz et al., 2008). The draft genome was uploaded to a web-based bacterial comparative genome analysis and functional annotation MicroScope (www.genoscope.cns.fr/agc/microscope/home/index.php). Genomes uploaded here can easily be accessed and analyzed by everyone. To visualize and compare the genome with other published *Enterococcus mundtii* genomes at the time of analysis, G-view server (https://server.gview.ca/) was also used (Petkau et al., 2010). Annotations for COG were performed using an online server WebMGA (http://weizhong-lab.uscd.edu); (Wu et al., 2011) and Blast2Go (Conesa et al., 2005). Genomic islands and virulence/resistance gene were annotated by Island Viewer 3, a cohesive interface for computational identity and visuality of genomic islands (Dhillon et al., 2015). VirulenceFinder-1.5 server (https://cgE.cbs.dtu.dk/services/VirulenceFinder/), was used to find the existence of virulence genes in genome (Joensen et al., 2014). Mining of the whole genome of the strain was performed to find the bacteriocins gene/Proteins using BAGEL (http://bagel.molgenrug.nl). ResFinder-2.1 server (https://cgE.cbs.dtu.dk/services/ResFinder/) was used to check the presence of the antibiotic resistance genes (Zankari et al., 2012). PAI Finder (http://www.paidb.rE.kr/pai_finder.php?m=f) was used to detect the presence of pathogenicity islands (Yoon et al., 2014). Genome sequence was mined for the detection of prophages sequences via online server PHAST (http://phast.wishartlab.com) (Zhou et al., 2011). CRISPR-Finder (http://crispr.i2bc.paris-saclay.fr/Server/) was utilized for the presence of the CRISPR (Grissa et al., 2007).

## Results

### Identification, Physiochemical and Technological Characterization

**Figure 1:**
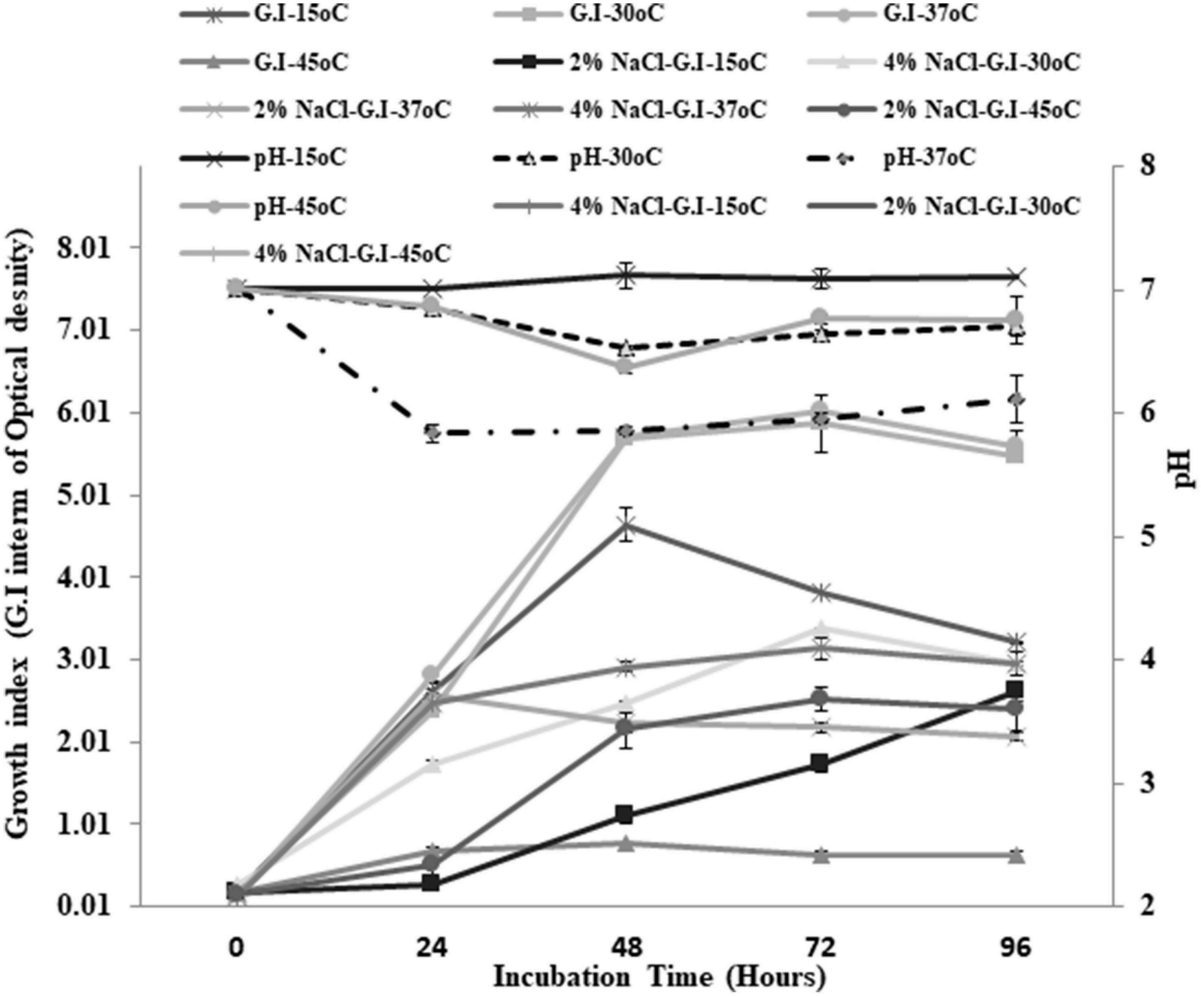
Growth at different temperatures and different NaCl concentrations for QAUEM2808 Y axis presents growth index, Y axis indicates incubation time, Z axis presents pH values, Ligands are above the figure.

Small, pinpoint, and yellowish colonies were observed on TSA plates. Gram staining revealed the presence of cocci in small and long chains. The strain showed negative results for catalase and oxidase enzymes. Different physiological parameters were studied for *Enterococcus mundtii* QAUEM2808. The strain showed maximum growth at 37°C among all tested temperatures. Increase in acidity of the medium (decrease in pH) was also maximum at the same temperature. Similarly, when growth of this strain at two different salt concentrations was studied under different incubation temperatures, the strain *Enterococcus mundtii* QAUEM2808 was found to be growing optimally at 4% NaCl concentration at 37°C (Figure 1). Clear zones were observed for positive isolates for the proteolytic, amylolytic and cellulytic activities. For cellulytic activity, clear zones were observed around the colonies after staining with Congo red and destaining with NaCl. For lipolytic activity, change in color of media indicated the positive results (Table 1).

**Table 1:** Classification and features of *Enterococcus mundtii* QAUEM2808.

**Property**	**Term**
Classification	Domain bacteria
	Phylum *Firmicutes*
	Class *Bacilli*
	Order *Lactobacillales*
	Family *Enterococcaceae*
	Genus *Enterococcus*
	Species *Enterococcus mundtii* Collins et al., 1986)
Strain code	QAUEM2808
Gram stain	Positive
Cell shape	Spherical or ovoid
Colony color	Yellow pigmented colonies
Motility	Non-motile
Sporulation	Non-sporulating
Temperature range	Mesophilic
Optimum temperature	Between 37 and 40°C
Salinity	Usually grow at 4–6% NaCl
Oxygen	Facultative anaerobe
Carbon source	Prefers disaccharides, but also ferment Lactose, sucrose, glucose, fructose
Mode of fermentation	Homolactic in glucose fermentation
Energy source	Chemoorganotrophs with fermentative metabolism
Habitat	raw milk, dairy products, Plant, intestine of human and animals, cow teats (Giraffa, 2003)
Biotic relationship	Symbiotic
Pathogenicity	None
Biosafety level	1
Isolation	Locally fermented milk product (dahi)
Geographic location	Islamabad, Pakistan
Sample collection time	28 August, 2013
Proteolytic activity	+
Lipolytic activity	+
Amylolytic activity	+

*The bacterium is classified and its features are described on the basis of laboratory experiments and the existing literature*.

### Evaluation of Antibiotic Resistance

The *E. mundtii* QAUEM2808 was sensitive against used antibiotics including Vancomycin, Erythromycin, Ciprofloxacin, Norfloxacin, Piperacillin, Tazobactactum, Doxycycline, Gentamycin, and Bacitracin except Penicillin for which the strain was found to be resistant. Further, we performed mining of the genome using online ResFinder-2.1 server (Zankari et al., 2012) at 80% threshold and 60% length for annotation, but did not find any antibiotic resistance or associated genes.

### Evaluation Biogenic Amine Production

**Figure 2:**
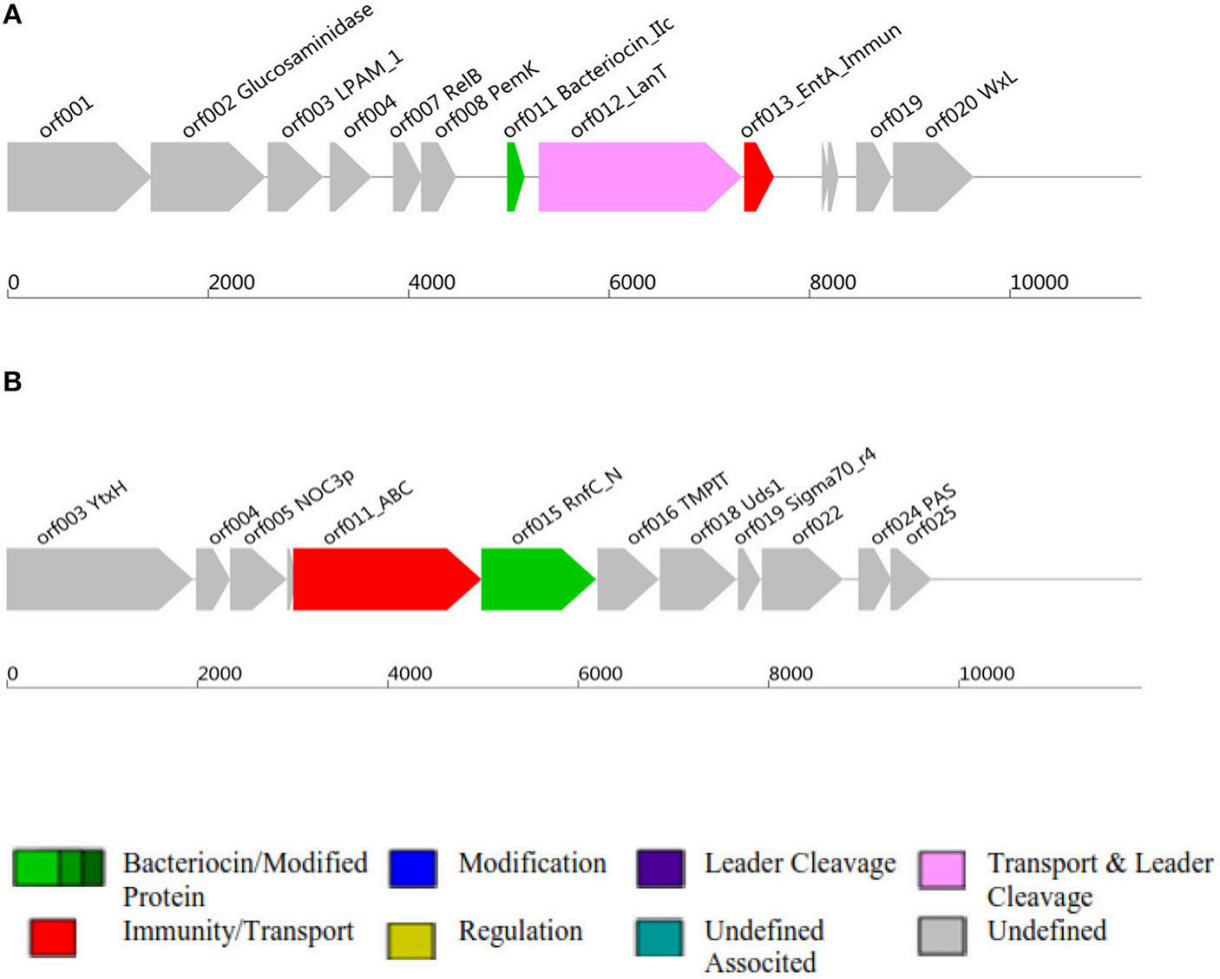
Class II and Class III bacteriocins ORF, Open Reading Frame; AOI, Area Of Interst; **(A)** indicates class II bacteriocin, **(B)** Class III bacteriocin.

The results for decarboxylation activity were recorded in terms of color changes (i.e., from yellow to purple) and quantification of decarboxylation activity was estimated through the intensity of purple color. Specific decarboxylation media and Trypticase soy broth supplemented with amino acids displayed the same decarboxylation activity (Figure 2). The *E. mundtii* QAUEM2808 showed moderate tyrosine decarboxylation activity at 37°C at 1, 2, and 3% NaCl concentration, while activity was negligible at higher NaCl concentrations (4 and 5%).

### Antimicrobial Activity and Bacteriocin Production

Antimicrobial activity of *E. mundtii* QAUEM2808 was evaluated against *E. coli* ATCC 10534 *S. aureus* ATCC 6538, *P. aeruginosa* ATCC 9027, and *L. monocytogenes* ATCC 13932. The strain exhibited activity against *E. coli*, forming a 12 mm zone of inhibition, *S. aureus* 18 mm, *P. aeruginosa* 18 mm, and for *L. monocytogenes* it was 14 mm. *In silico* results for the bacteriocin are presented in Table 2. Protein ID represents the bacteriocins ID present in the genome in the form of ORF, open reading frame. AOI stands for area of interest, which is that part of nucleotide sequence which is analyzed in more detail during the identification of bacteriocin encoding genes. Start position represents the ORF starting location and the location of the gene in AOI the bacteriocin at start and end point in AOI. Annotation of bacteriocins are performed by PFAM which is integrated into BAGEL and PFAM. Domain ID is given in the Table 2 and depicted in Figure 2. Results for the b can also be accessed by following the link to MicroScope (http://www.genoscope.cns.fr/agc/microscope/metabolism/domainviewer.php?id=&prog=Cluster&ASC_id=36804).

**Table 2:** Bacteriocins produced by *Enterococcus mundtii* QAUEM2808 Mundticin and enterolysin.

**Type**	**Protein ID**	**Area of Interest ID (Dawid et al., 2007)**	**AOI start position**	**Position of the gene in AOI**	**Strand**	**PI**	**Length**	**PFAM name**	**No of Cys and Thr**	**Lantibiotic PFAM domain**	**Blast hit in bacteriocin II database**	**Blast hit in bacteriocin III database/Homology (*P*-value)**
Sactipeptides	AOI1;orf006	AOI_1	28,260	3,024–3,281	+	9.1	85		Cys = 1 SerThr = 8	primary_pfams		
ClassIII	AOI2;orf015	AOI_2	48,001	5,000–6,205	+		401	PF13375.1 [4.3e-05]				enterolysin_A[9e-27]
ClassII	AOI1;orf011	AOI_1	1,942	5,000–5,176	+	10.3	58	PF10439.4 [0.00021]	Cys = 2 SerThr = 7	PF01721.13 PF10439.4	Mundticin_ATO6 [1e-36]	

*Protein ID means the bacteriocins ID present in the genome in the form of Open Reading Frame. AOI, Area Of Interest, the part of nucleotide sequence analyzed in more detail during identification of bacteriocin encoding genes; start position, ORF start position, position of gene; the bacteriocin start and endpoint in AOI. Link to access the results (http://www.genoscope.cns.fr/agc/microscope/metabolism/domainviewer.php?id=&prog=Cluster&ASC_id=36804)*.

### Folate Production

**Figure 3:**
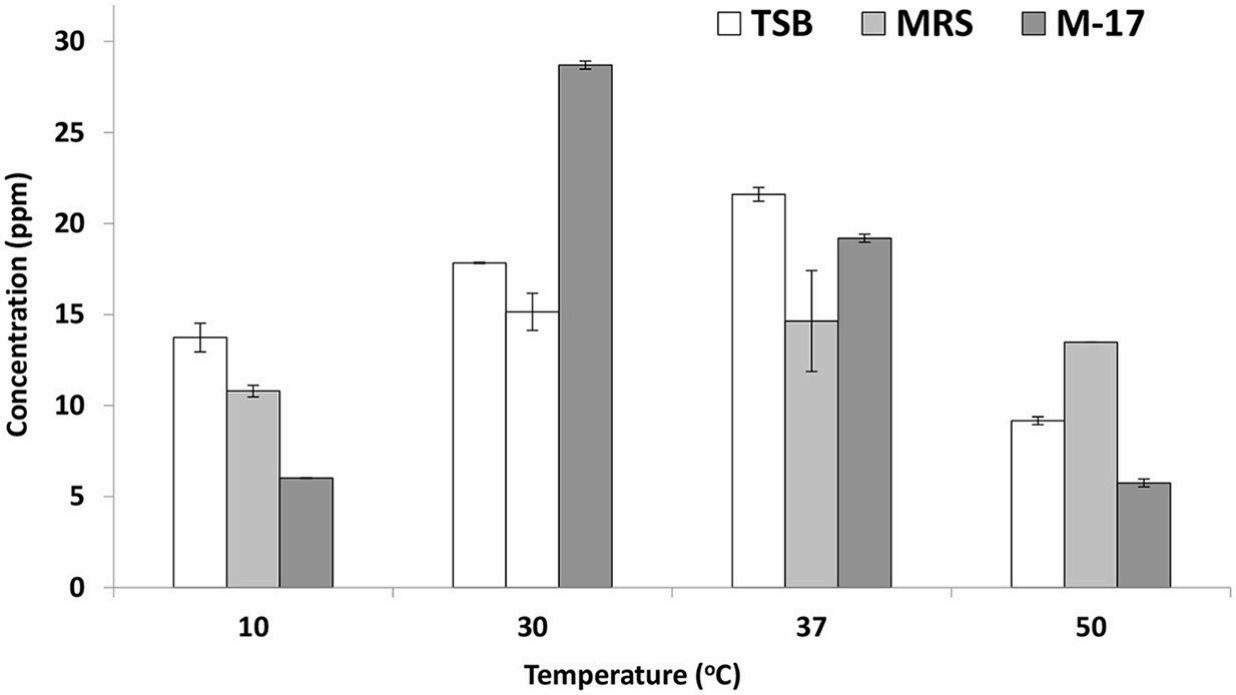
Folate production by *Enterococcus mundtii* QAUEM2808 at different temperatures. It presents folate concentration in ppm in different growth media at different temperatures.

On trypticase soy broth, *E. mundtii* QAUEM2808 showed folate activity when incubated at 37°C temperature. At all other temperatures, its activity was poor due to reduced growth. Growth and folate production were minimum at the highest and lowest incubating temperatures in broth media (Figure 3).

### The *in vivo* Safety Assessment

#### Hematological Analysis of Mice

The Balb/c has been recognized to have robust Th2-type immune response (Kong et al., 2016). However, in our conditions, blood CP (complete picture) of control and experimental group showed no abnormal changes such as increase in immune cells which corresponds to infectious state of the body, thus supporting the safety of administered *E. mundti* QAUEM2808 (Data not shown).

#### Histological Analysis of Liver and Small Intestinal Sections of Mice

**Figure 4:**
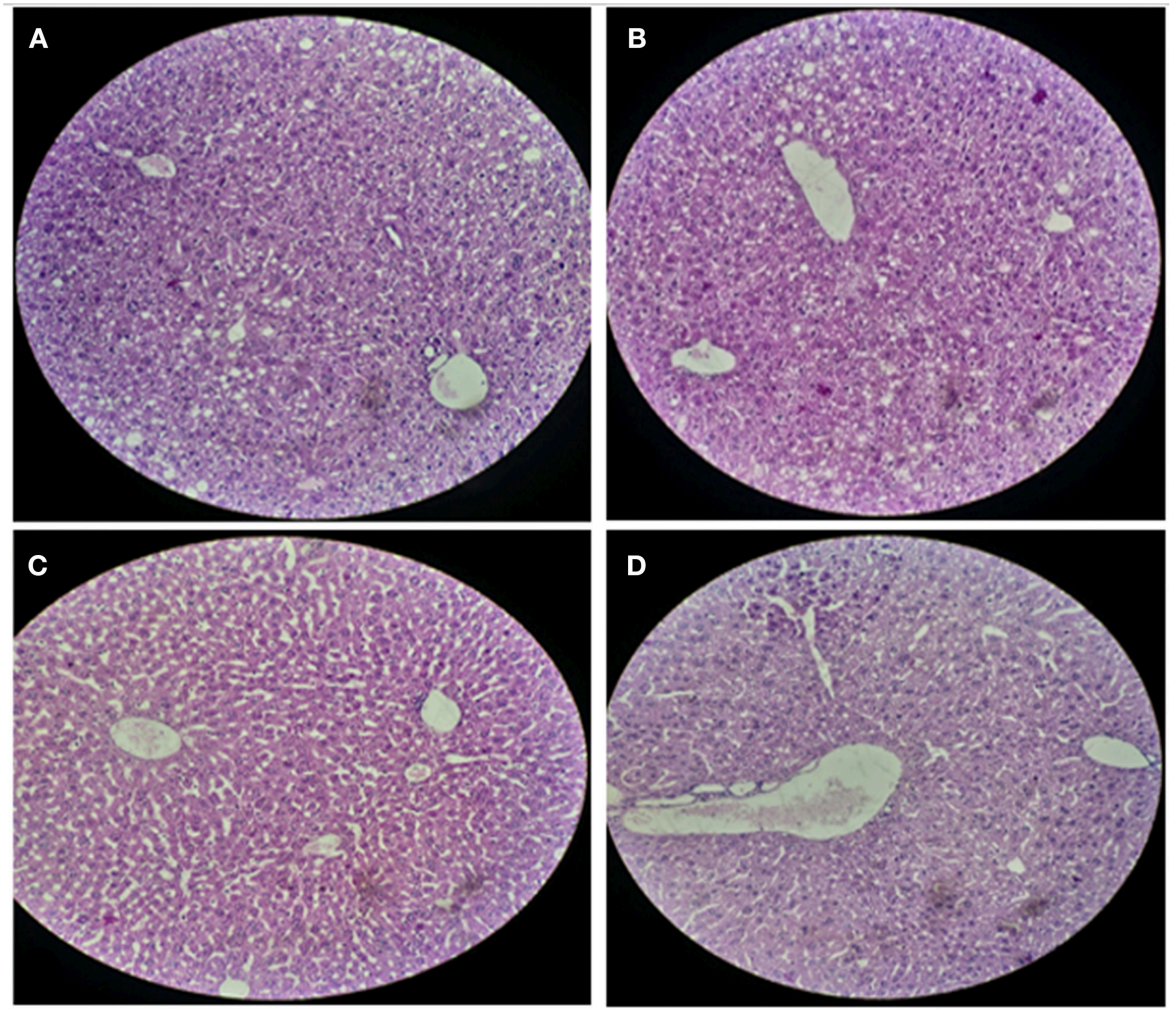
Liver sections of mice at 20X microscope. **(A,B)** sections, when there is no intake of any probiotic or experimental bacterium, **(C,D)** sections, when micro *Enterococcus mundtii* QAUEM2808 was administered.

**Figure 5:**
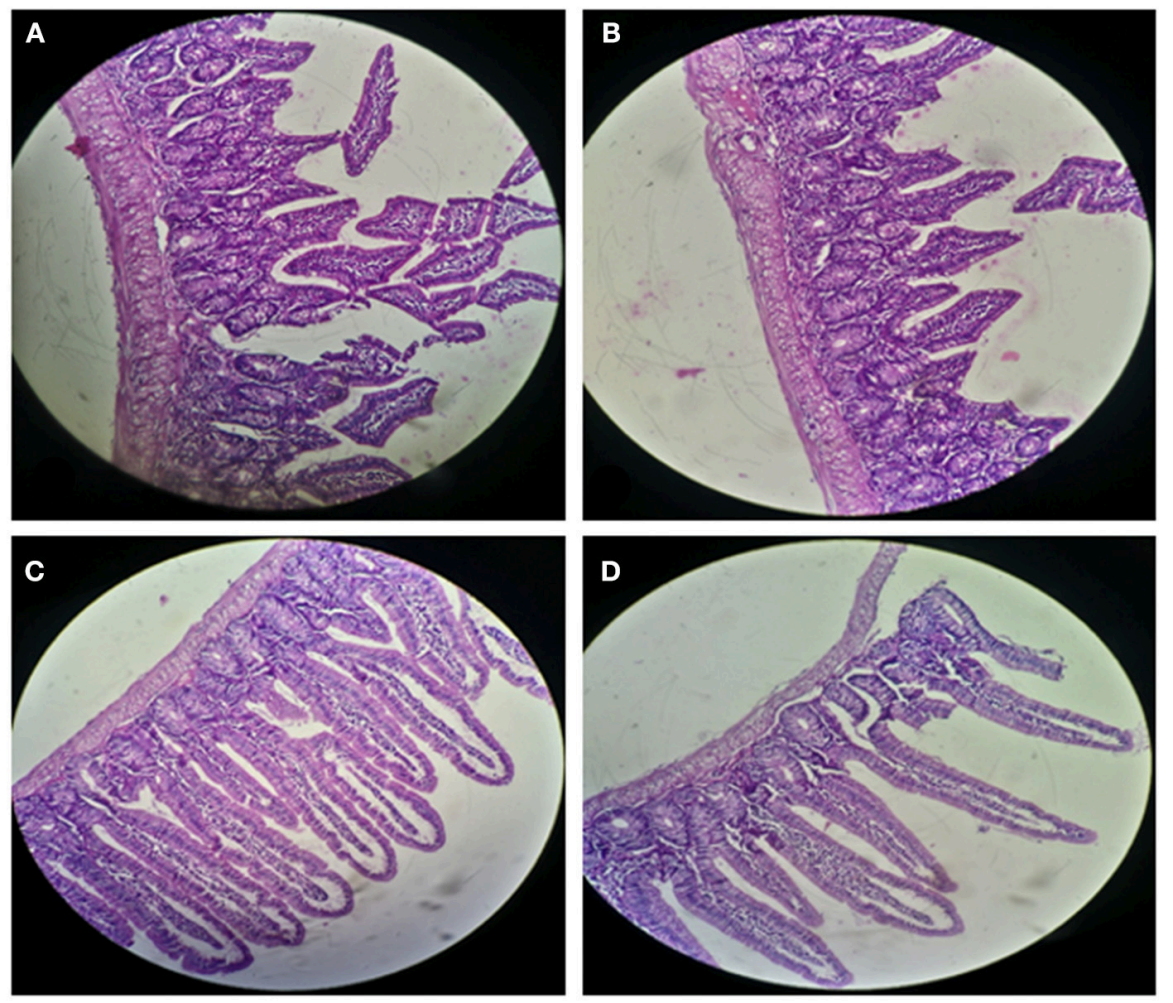
Small Intestinal sections of mice at 20X microscope **(A,B)** negative control, **(C,D)** with *Enterococcus mundtii* QAUEM2808 administration.

**Figure 6:**
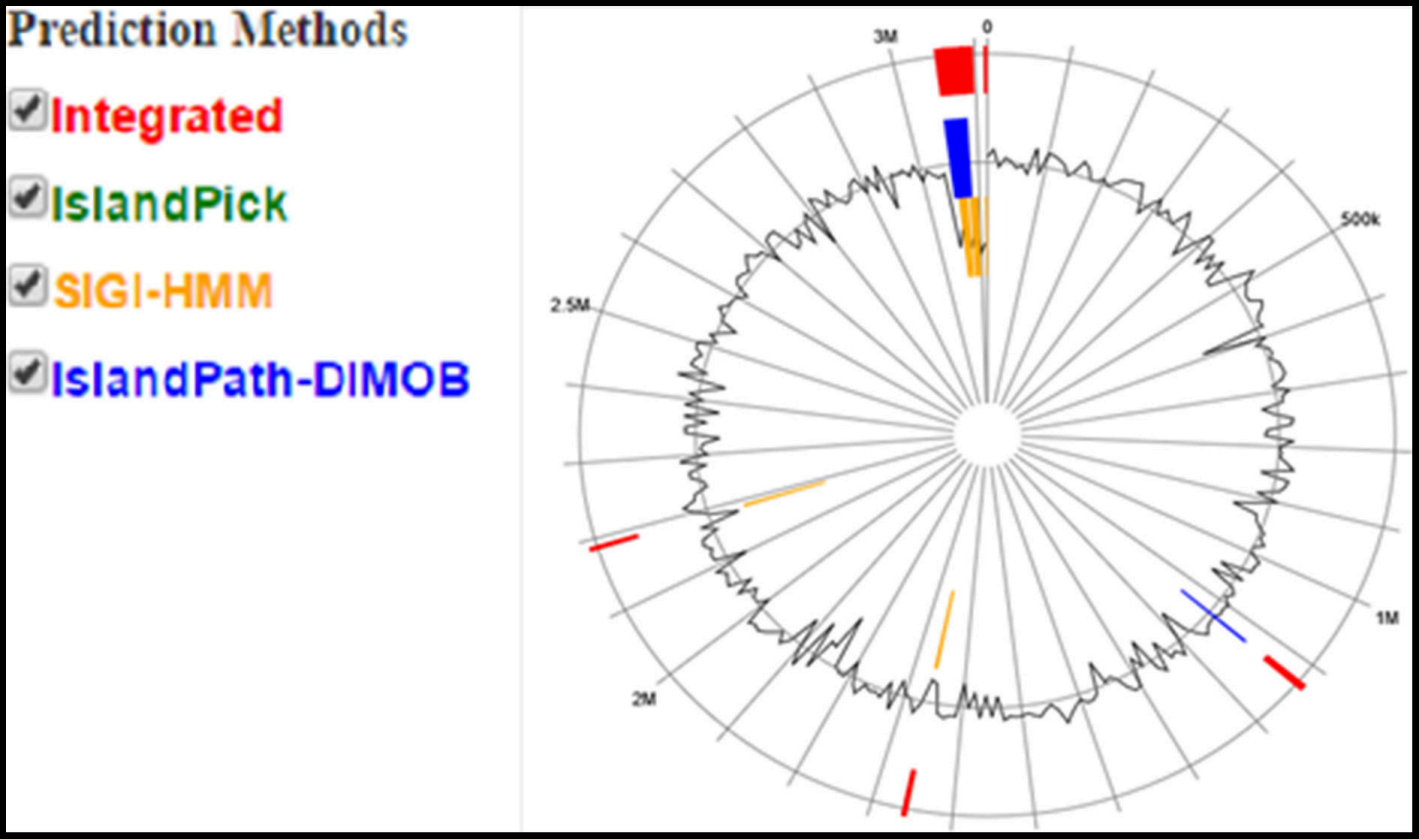
Circular plot of *Enterococcus mundtii* QAUEM2808 genome islands Color scheme used in IslandViewer such as green for IslandPick, orange for SIGI-HMM, red for integrated prediction methods, http://www.pathogenomics.sfu.ca/islandviewer/results/16275/?token=rhXpqAaWROfmhio87Io6sl.

For histological analysis, mice were dissected and parts of the liver and small intestine were picked, sectioned and stained with hematoxylin and eosin and observed under compound microscope (Olympus, Japan). The liver parenchymal architecture was preserved. The hepatocytes were arranged in cords of 1–2 cells thickness. The portal tracts showed mild lymphocytic infiltrate but there was no evidence of fibrosis, necrosis or malignancy in the slides examined which supports the hepatocellular safety of *E. mundtii* QAUEM2808. Histological slides presented no morphological changes related to the control feeding treatment (Figure 4). The crypt structure was intact, all of the intestinal sections appeared normal and healthy when examined; no signs of detachment and necrosis in enterocytes, widening in lamina propria or necrosis were observed (Kristiansen et al., 2011). No evidence of mucosal damage, necrosis, granuloma or malignancy were found in the intestinal sections examined (Figure 5). Genomic islands and virulence/resistance gene annotations were projected using IslandViewer-3 http://www.pathogenomics.sfu.ca/islandviewer/ (Dhillon et al., 2015). The inner circular graph showed GC content deviation. Regions highlighted in red, blue, green, and yellow illustrate predicted islands. Starting and ending position of predicted islands and their size along with prediction methods used are given in Figure 6.

### Phylogenetic Tree

**Figure 7:**
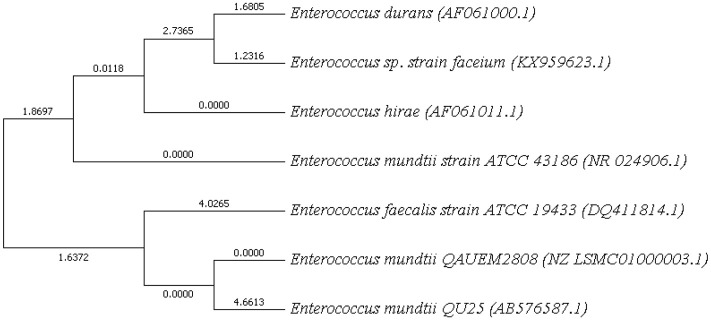
16s rRNA based Phylogenetic tree of *Enterococcus mundtii* QAUEM2808.

NCBI blast was used and strains with maximum score identity were selected. Their 16 s rDNA sequences were retrieved from NCBI. MEGA7 was used for phylogeny finding. Boost trap method was used with score of 600 (Figure 7). This analysis involved sequences from *Enterococci* species available at NCBI. BLAST was performed for *E. mundtii* QAUEM2808, *E. mundtii* ATCC *882* whole genome shotgun sequence and *E. mundtii* QU25. Neighbor joining tree was obtained, the relation of *Enterococcus mundtii* QAUEM2808 with other strains is also available at NCBI.

### Genome to Genome Distance

Genome-to-Genome Distance was calculated by GGDC calculator (http://ggdc.dsmz.de/background.php). Distances (Table 3) were conditioned by using three different formula from the group of HSPs. MUMs were obtained by relating each pair of the genome via selected software. These distances were converted to the values comparable to DNA-DNA hybridization (DDH) utilizing a Generalized Linear Model (GLM) conditioned from an observed position dataset containing real DDH values and genomes sequence. Model-based sureness pauses were stated in the square brackets but may also get through bootstrapping. Logistic regression (pecial type of GLM) was used for reporting the probabilities that DDH is ≥70 and ≥79%. Percent G+C contents can't vary by >1 inside a single specie but can vary by <=1 among different species (Meier-Kolthoff et al., 2013).

**Table 3:** GGDC calculator results DNA-DNA hybridization (DDH), Bootstrap C.I (C.I Confidence Interval).

		**Formula 1**					**Formula 2**
**Query genome**	**Reference genome**	**DDH**	**Model C.I**.	**Bootstrap C.I**.	**Distance**	**Prob. DDH ≥ 70%**	**DDH**
EM2808	*Enterococcus mundtii QU25*	83.1	[80.1–85.8%]	82.2–83.8%	0.244	94.51	0.07
EM2808	*Enterococcus mundtii* ATCC 882	68.4	[65.2–71.4%]	67.4–69.5%	0.3599	71.32	0.09
EM2808	*Enterococcus mundtii* crl1656	21.2	[18.8–23.7%]	21.1–21.2%	0.9664	0.01	1.02
EM2808	*Enterococcus mundtii* ATCC 882	81.4	[78.3–84.1%]	80.6–82.2%	0.2583	93.13	0.09

The draft genome of *Enterococcus mundtii* QAUEM2808 strain contained 2,993,664 bp circular type chromosome and average G+C content of chromosome was 38.5%. The genome was predicted to have 2,707 coding sequences (CDS), 47 RNAs. The genome was deposited in GenBank “NCBI Prokaryotic Genome Annotation Pipeline” under accession number PRJNA311247. General genome statistics are given in Table 4.

**Table 4:** Genome Statistics of *Enterococcus mundtii* QAUEM2808 (Obtained from RAST server).

Genome	*Enterococcus mundtii* draft genome
Domain	Bacteria
Taxonomy	Bacteria; *Enterococcus mundtii*
Size and G+C	2,993,664 bp, 38.5%.
Number of subsystems	326
Number of coding sequences	2,707
Number of RNAs	47

### Genome Features

#### Circular Map of *Enterococcus mundtii* QAUEM 2808

The circular map of *Enterococcus mundtii* QAUEM 2808 with putative function was generated by Gview server (*https://server.gview.ca/)*. Comparative genomic studies between *Enterococcus mundtti QU25* (NC_022878), *atcc882* (NZ_KB946218), *CRL1656* (NZ_AFWZ01000001), *atcc882* (NZ_ASWC00000000) whole genome shot gun, *CRL35* (NZ_JDFT01000001.1) were performed using G-view server (https://server.gview.ca/) (Figure 9). The results obtained by G-view server in the form of circular and linear map are shown below http://www.ncbi.nlm.nih.gov/genome/genomes/11638.

### Prophage Sequences and CRISPRs—Cas Systems

Both partial and complete prophages were distinguished via online prophage search and annotation pipeline PHAST.27. Only one incomplete prophage of 7.036 kb with 35.42% GC was detected. The size of the intact prophage was too small when compared to the intact prophages detected in all the bacterial genomes, isolated from bacteria of dairy origin. Clustered Regulatory Interspaced Short Palindromic Sequences were annotated with CRISPR-Finder web server (Grissa et al., 2007). Contig_40 contains a possible CRISPER between sequences 103,287–103,407 of 120 bp with one spacer.

### Metabolic Network

The metabolic Pathway/Genome Database (PGDB) was created computationally with KEGG Metabolic pathway in RAST Annotation server relied on annotated EC values and a modified enzyme name record file (Table 5).

**Table 5:** Metabolic subsystems features and counts (the table is generated using RAST server).

**Subsystem features**	**Counts**
Cofactors, vitamins, prosthetic groups, pigments	76
Cell wall and capsule	118
Adhesion	2
Toxins and superantigens	0
Bacteriocins, ribosomally synthesized antibacterial peptides	7
Resistance to antibiotics and toxic compounds	31
Virulence, disease and defense—no subcategory	0
Invasion and intracellular resistance	13
Potassium metabolism	9
Photosynthesis	0
Miscellaneous	22
Phages, prophages, transposable elements, plasmids	2
Membrane transport	52
Iron acquisition and metabolism	22
RNA Metabolism	117
Nucleosides and nucleotides	94
Protein metabolism	208
Cell division and cell cycle	42
Motility and chemotaxis	1
Regulation and cell signaling	34
Secondary metabolism	3
DNA metabolism	114
Fatty acids, lipids, and isoprenoids	73
Nitrogen metabolism	0
Dormancy and sporulation	6
Respiration	23
Stress response	63
Metabolism of aromatic compounds	2
Amino acids and derivatives	179
Sulfur metabolism	9
Phosphorus metabolism	30
Carbohydrates	443

## Discussion

The use of microorganisms for fermentation is well-known since the Neolithic age. Many bacterial strains have been used industrially for hundreds of years for the preservation of food, medication, and other valuable products. Bacteria during fermentation produce a variety of metabolic products which can give the food product several properties related to preservers, texturizers, stabilizers, flavoring and coloring. The enterococci constitute a complex and important group of bacteria occupying multiple ecological niches including human gut microbiome, fermented foods and plants. Usually it is attributed to their capacity to tolerate heat treatments and hostile ecological situations. They also have a significant industrial role in the production of meat and dairy products such as improvement of the aroma or ripening of the cheese. They can also produce bacteriocins against foodborne pathogens. Due to their role in the ripening, flavor refinement and anti-bacterial peptides production, enterococci with desired technical and metabolic characteristics have been proposed to be used as starter culture, adjunct culture or co-cultures in various fermentations. Because of the risks for the involvement of enterococci in spreading resistance to antibacterials and production of toxic agents like biogenic amines, it is compulsory to assess the safety of the strain isolated from foods of various geographical areas, particularly when they are intended to be used as starters or they have related roles in the fermentation and/or ripening of the traditional foods. In this perspective, the initiative of our study was to characterize enterococci strain isolated from the indigenous fermented milk product dahi, due to specific features i.e., occurrence of virulence agents, antibiotics resistance and the production of biogenic amines.

**Figure 8:**
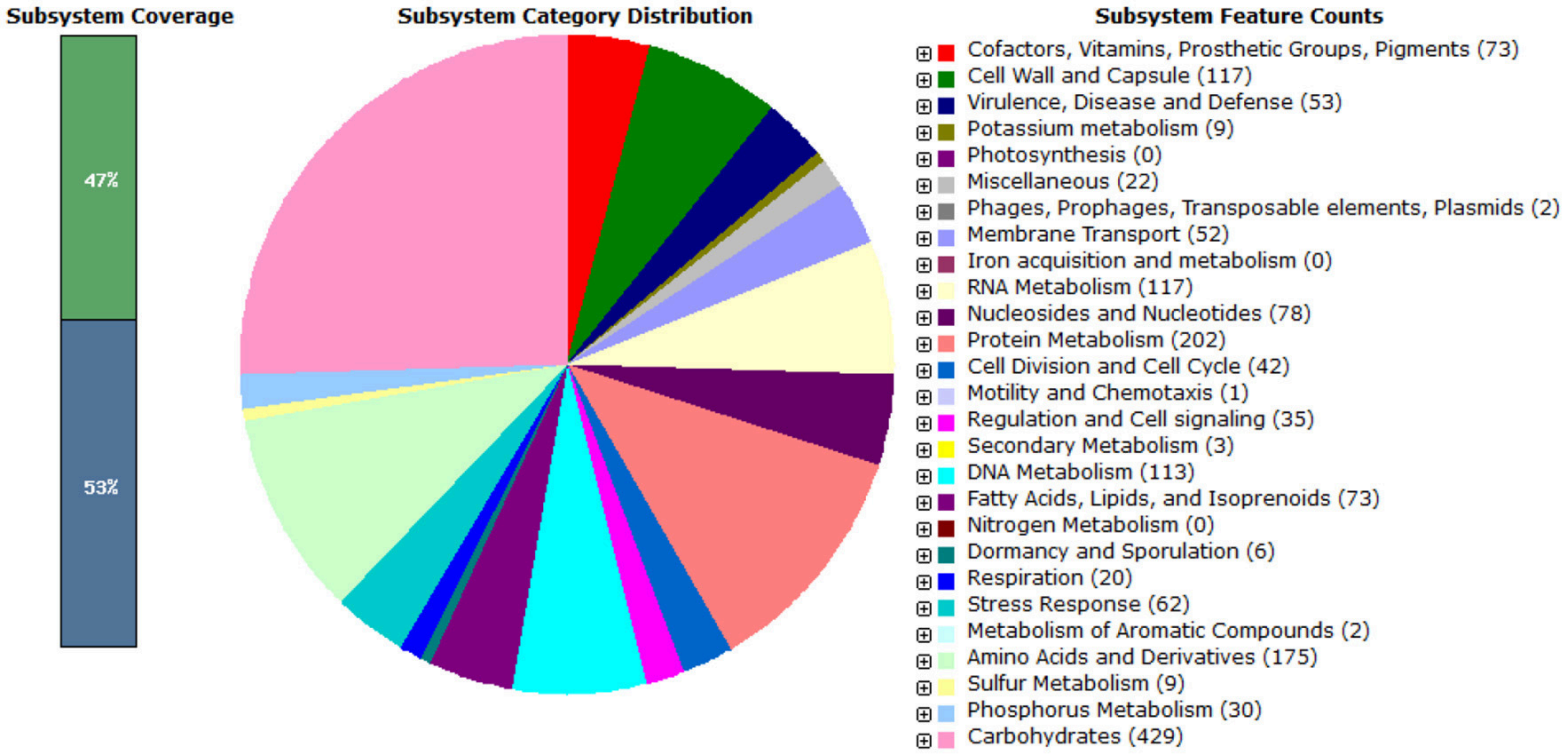
Pie chart showing functional genome of *Enterococcus mundtii* QAUEM2808. The genes are predicted using FIGFams system integrated within RAST online server.

In the present study, *Enterococcus mundtii* QAUEM2808 showed maximum growth at 37°C. Hence, it was grouped as mesophilic. When two different NaCl concentrations were used, the strain showed little growth at 2% NaCl concentration, while at 4% NaCl concentration the growth rate was maximum for the strain *Enterococcus mundtii* QAUEM2808 (Fisher and Phillips, 2009). The *Enterococcus mundtii* QAUEM2808 showed proteolytic, lipolytic, amylolytic and cellulolytic activities in the respective media. Proteolytic and lipolytic activities are responsible for aroma and flavor development by food grade enterococci (Manolopoulou et al., 2003). RAST functional annotation also showed the presence of these genes as shown in Figure 8.

**Figure 9:**
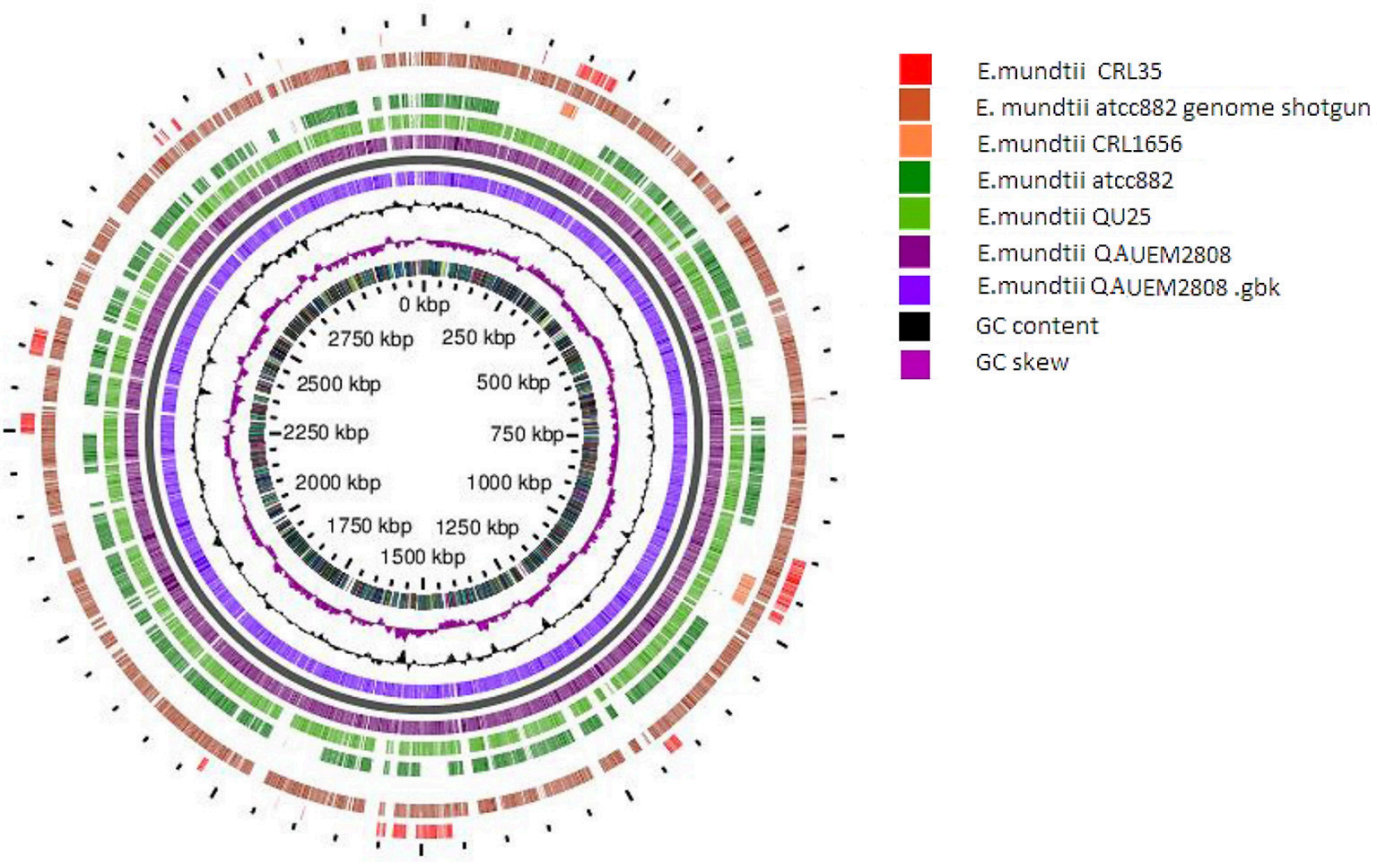
Circular map of *E. mundtti* QAUEM2808 genome and Comparative genomics. comparison with other *E. mundtii* genomes available at the time of analysis result, https://server.gview.ca/job/D10220292573E3CDA8C26C6756F807D0/results.

Being a salt tolerant strain, it could be a good candidate for the cheese ripening process. It was found that the *Enterococcus mundtii* QAUEM2808 was sensitive to commonly used antibiotics including Vancomycin, Ciprofloxacin, Norfloxacin, Tazobactactum, Doxycycline, and Bacitracin except Penicillin for which the strain was found to be resistant. These results are similar to the finding that most of the food reservoir *Enterococcus* species are less resistant to the commonly used antibiotics while some of the *Enterococcus faecium* strains were found resistant to the Penicillin in an early report (Kaçmaz and Aksoy, 2005). Resistance to penicillin has first been documented in 1946, while genes responsible for Pencillin resistance are primarily involved in the repair mechanism of the cell wall and are responsible for the stability of the organism (Barber and Rozwadowska-Dowzenko, 1948; Brooks et al., 2006). Unlike clinical isolates of *Enterococci*, our strain is found to be sensitive to vancomycin (Praharaj et al., 2013; Biswas et al., 2016; Rengaraj et al., 2016) justifying its presence in food matrix. When ResFinder-2.1 was used to search out antibiotic resistance genes present in the genome of *E. mundtii* QAUEM2808 with minimum 50% and maximum 80% similarity, the server did not detect any putative resistant gene (Zankari et al., 2012).

The *E. mundtii* QAUEM2808 showed moderate tyrosine decarboxylation activity at 37°C at 1, 2, and 3% NaCl concentration, while activity was negligible at higher NaCl concentrations (4 and 5%). Usually high concentrations of salt are used to reduce spoilage microbes from the fermented foods, during the process of avoiding food intoxication and spoilage (Linares et al., 2012). This reduction of microbes is due to the addition of salt and results in the reduction of biogenic amines producers. In cheese *Enterococcus* strains are the main cause of biogenic amine production but with the addition of 5% NaCl to the *Enterococcus* inoculated milk, very low concentrations of tyramine and 2- phenyl ethylamine were reported (Gardini et al., 2001). The folate production was observed in *E. mundtii* QAUEM2808. Maximum folate production was observed at 37°C in all media which indicated that growth of *Enterococcus mundtii* QAUEM2808 was optimum at this temperature. Overall in the study, MRS broth was found to be the best among the three media tested for folate accumulated in cultures of *E. mundtii* QAUEM2808 (Lin and Young, 2000). All the media had shown disparate behavior at other temperatures. Results indicated that *Enterococcus mundtii* QAUEM2808 grew well in a temperature range of 30–37°C and showed poor growth at extreme of temperatures i.e., 10 and 50°C as reported previously (Barbosa et al., 2016). Cornwell had revealed that lactic acid cultures not only synthesized folate but also consumed it. This could explain why at both maximum and minimum temperatures, *Enterococcus mundtii* QAUEM2808, instead of producing folate, consumed it for its survival (Patel et al., 2013).

Folate biosynthetic gene cluster of *Enterococcus mundtii* QAUEM2808 as predicted by KEEG pathway tool integrated with MicroScope (which can be accessed by following the link: http://www.genoscope.cns.fr/agc/microscope/metabolism/keggtabmap.php?Pid=00790&Beg=0&End=1&S_id=9820) was different from organisms reported earlier showing variations from other genus of lactic acid bacteria. The presence of genes *folC* and *folA* for enzyme polyglutamyl folate synthetase and dihydrofolate reductase enzymes indicated that folate production pathway is incomplete. It is assumed that this bacterium can grow in association with other indigenous microorganisms and work synergistically with them. The *folC* is among the common enzymes that have a role in biosynthesis of the folate. As reported in literature, the overexpression of *folC* increases the production of folate five times in *L. lactis* indicating the opportunity to enhance folate production in *Enterococcus mundtii* QAUEM2808. The presence of aminodeoxychorismate lyase *pabC* gene depicted the formation of PABA from chorismate which is one of the precursors of folate.

Several studies support the hypothesis of the production of folate by lactic acid bacteria. It is reported that industrial starter cultures such as *Lactococcus lactis* and *Streptococcus thermophiles* are the native producers of folate. For this reason, some fermented dairy products are supposed to have higher folate level than non-fermented products (Jones and Nixon, 2002; Aryana, 2003; Verwei et al., 2003; Jägerstad et al., 2004; Kariluoto et al., 2006). Different species of *Bifidobacteria* have the ability to produce folate (Rad et al., 2016). Among the enterococcus group, *Enterococcus faciem* is reported as a folate producer. It is reported that milk fermented with mixed cultures has high contents of folate (Crittenden et al., 2003).

The safety of microorganisms is an important factor while considering them as consumable food, and besides their beneficial role, they may also have adverse effects when administered live in a complex body metabolism (Pavan et al., 2003). For the last few decades the use of food cultures has increased in a significant manner, followed by the increase in safety guidelines by regulatory authorities ensuring the health and protection of consumers (Laulund et al., 2017). Body immune responses and histological profiles are reliable parameters to investigate the impact and safety of any administered agent. In this regard hematological and histological analysis were carried out, together with the data presented here, follow the safety of *Enterococcus mundtii* QAUEM2808 in balb/c mice. No harmful effect of this strain was observed on overall health. WBCs being active members of the cellular immune response toward any foreign agent of pathogenic category, are very sensitive to the presence of toxic agents in the body (Hodyl et al., 2008). RBCs and platelets count, along with other parameters in Blood picture, also deviate from the standard when the body gets infected by any pathogenic organism due to its abnormal metabolites and immunogenic substances (Benković et al., 2012). In our study WBCs, along with other immune cellular components, were within recommended reference ranges in the Blood CP of experimental group featuring *Enterococcus mundtii* EM2808 administration, which is a positive sign for its safety supporting.

Liver and intestine are exposed to all that is present in the gut as well as in the blood and are involved in the detoxification of harmful substances produced by invasive or pathogenic agents in the body, especially when entering through the oral route (Chodorowski et al., 2007). Therefore, the histological analysis of these organs is significant in order to comment responsibly about the compatibility and tolerance of the administered agent in the body. Abnormal Tissue morphology, distorted cellular adhesion, pigmentation and cellular inflammation lead to the loss of tissue function and it depends upon the extent of pathological aspects a tissue faces when encountered with toxic metabolites of any foreign organism (Sherlock and Dooley, 2008). Probiotics, the safe group of microorganisms, are known to prevent and maintain the hepatic functions and physiology in a healthy manner (Ewaschuk et al., 2007); as per the conclusion of our findings, *Enterococcus mundtii* QAUEM2808, which is emerging as a nonpathogenic food grade strain, also preserved the normal structure and physiology of mice as the tissue structure in the histological slides was preserved without any evidence of pigmentation or inflammation in hepatocytes and in overall liver sections examined.

The results of the present study demonstrated that *Enterococcus mundtii* QAUEM2808 was safe when close to the dosage of 2 × 10^9^ cfus per mice per day in a 90-days trail. It was well-mounted and no adverse effects were seen on the growth, blood components and vigorous organs of the experimental mice. The normality of parameters inspected above was very sensitive and significant to the safety of any foreign agent, therefore it was successfully concluded that the use of *Enterococcus mundtii* QAUEM2808 didn't have any harmful effect on the internal organs and systemic functions in treated mice (Shokryazdan et al., 2016). Virulence/resistance gene annotations results predicted that there is no pathogenic gene in *Enterococcus mundtti* QAUEM2808 genome. These results are comparable with those of (Repizo et al., 2014) who observed that *Enterococcus mundtii* CRL1656 does not contain the most significant virulence factors present in the clinical isolates of enterococci. Online prophage search and annotation showed that there was only one incomplete prophage of 7.036 kb with GC% 35.42. The size of the intact prophage was too small to be compared to the intact prophages detected in all the bacterial genomes, isolated from bacteria of dairy origin. A comprehensive analysis was done by Bonacina et al. (2016). Majority of the genomes published in NCBI contained prophages (Canchaya et al., 2003). The low number of prophages present in a genome is a sign of safety and stability as the genome of GRAS *Lactococcus lactis* contained six prophages (Canchaya et al., 2003). Analysis of CRISPER showed that Contig_40 contain a possible CRIPER between sequences 103,287–103,407 of 120 bp with one spacer. Tdim et al. found that resistance to antibiotics and ownership of CRISPR loci are inversely related (Tedim et al., 2015). Both the presence of CRISPR and the sensitivity to daily used antibiotics confirmed the safety of *Enterococcus mundtii* QAUEM2808. *In silico* analysis showed that *Enterococcus mundtii* QAUEM2808 carried class II and III bacteriocins. Class II bacteriocins are produced by streptococcal species to prevent closely related gram positive bacteria. This bacteriocinogenic member of lactic acid bacteria has been proposed as a probiotic strain which can stop mastitis in cows (Espeche et al., 2009). Bacteriocins are antibacterial short peptides which are mostly synthesized by bacteria ribosomally. Bacteriocins are small in size and when no similarity to known proteins is found then these ORFs are omitted from annotations due to their small size (de Jong et al., 2010). The BAGEL is the only available annotation tool which could be used to discover a new bacteriocins as it ensures that no putative bacteriocins is missed (de Jong et al., 2010). BAGEL classifies the bacteriocins based on similarity with identified bacteriocins, presence of motifs, features associated to a specific class and context gen*E*. BAGEL is used for class specific mining of genomes for bacteriocins and it also classifies these putative bacteriocins into their specific classes viz, Class I, Class II, and Class III. Class I consists of lantibiotics which can be further sub classified, and class II are non-lantibiotics which can also be sub classified into groups A, B, C, and D. Class III consists of relatively large antimicrobial proteins (Dong et al., 2010).

Microorganisms present in a particular food are only considered safe and desired when their presence in food does not effect overall quality and quantity of the food or food products but improves the quality, quantity or texture of the food and food products. Their presence increases food demand for consumers and they have no side and adverse effects on the consumer's well-being and health. As the strain *Enterococcus mundtii* QAUEM 2808 is isolated from the indigenous fermented milk product, dahi is considered natural to this product. The indigenously fermented milk product dahi is known for its antipathogenic activity. That is due to low pH and anti-bacterial peptides. This bacteria, as shown by experiments, have acidification ability along with bacteriocin production and antipathogenic activities. Proteolytic, lipolytic, amylolytic, and cellulytic properties of the bacterium are considered indicators for the improvement in quality of the dairy products in terms of texture, aroma and flavor. *In vivo, in vitro* and genomic studies of the bacterium confirmed its safety if used in food and food products. The bacterium has the ability to produce folate, hence dahi having *E. mundtii* QAUEM 2808 can be used as functional food.

## Ethics Statement

This study is approved by the ethics committee of Quaid-i-Azam University Islamabad Pakistan.

## Author Contributions

FN isolated and characterized the strain. MK did *in-silico* analysis and drafted the manuscript. AJ checked the strain for cytotoxic effect in mice. SB did genomic analysis. IA, NA, and MA helped in the interpretation of the results. MI supervised the study, critically reviewed and finalized the manuscript.

### Conflict of Interest Statement

The authors declare that the research was conducted in the absence of any commercial or financial relationships that could be construed as a potential conflict of interest.
